# Some Exploded Theories about Bullet Wounds

**Published:** 1900-09-15

**Authors:** 


### SOME EXPLODED THEORIES ABOUT BULLET WOUNDS.

Several theories in regard to bullet wounds seem, according to Mr. Clinton Dent, to have been finally exploded by recent experience. One of these is that the heating of the bullet sears the track of the wound and so destroys any germs that might lodge, thus favouring immediate union and preventing septic complications. As a matter of fact, he says, bullets fired from modern small-bore rifles are in the first place usually sterile, and in the next they are not heated; but even if they were white hot the time that they occupy in passing through the body is so extremely short that no cauterising effect could possibly take place. A bullet making a flesh wound of six or eight inches long, at a range of 800 or 1,000 yards, occupies somewhere about one three-thousanth part of a second in going through. Even when a bullet strikes a bone and lodges and thus has both its forward movement and that of rotation arrested, no cauterising effect is found in the neighbourhood, its energy being really dissipated in other directions. Then again, the so-called hydrodynamic or hydraulic theory has been held to explain the extending or explosive effects of rifle bullets on animal tissues. Those who believe this theory look upon a limb as a closed vessel containing fluid, and account for the destruction which is undoubtedly seen sometimes at short range as due to the enormous increase of pressure caused by the ingress of the bullet on the incompressible contents of the part hit. This theory, though abandoned for the most part in regard to the greater part of the body, is still considered by some to hold good for penetrating wounds of the skull. Experiments, however, do not bear out this hydraulic view, and the explosive or expanding effects are now held to be due to the energy of the bullet, which radiates out in lines from the long axis of the bullet track. Another notion that has gone the same way as the hydraulic theory is the projectile air theory. This imagines that a cushion of highly compressed air is carried in in front of the bullet, and produces the explosive effect. This is an old theory which has been lately revived, but it seems to have no basis in fact, and the same may be said of the old idea about what were called wind contusions from large projectiles. These are no longer believed in. They disappeared when round shot ceased to be used, the fact being that round shot would hit a part and cause the most grievous injury to the deeper tissues without showing any mark whatever.

